# Integrating Ecological and Engineering Concepts of Resilience in Microbial Communities

**DOI:** 10.3389/fmicb.2015.01298

**Published:** 2015-12-01

**Authors:** Hyun-Seob Song, Ryan S. Renslow, Jim K. Fredrickson, Stephen R. Lindemann

**Affiliations:** ^1^Biological Sciences Division, Earth and Biological Sciences Directorate, Pacific Northwest National LaboratoryRichland, WA, USA; ^2^Environmental Molecular Sciences Laboratory, Pacific Northwest National LaboratoryRichland, WA, USA

**Keywords:** microbial communities, microbial ecology, resilience, resistance, robustness, stability, networks

## Abstract

Many definitions of resilience have been proffered for natural and engineered ecosystems, but a conceptual consensus on resilience in microbial communities is still lacking. We argue that the disconnect largely results from the wide variance in microbial community complexity, which range from compositionally simple synthetic consortia to complex natural communities, and divergence between the typical practical outcomes emphasized by ecologists and engineers. Viewing microbial communities as elasto-plastic systems that undergo both recoverable and unrecoverable transitions, we argue that this gap between the engineering and ecological definitions of resilience stems from their respective emphases on elastic and plastic deformation, respectively. We propose that the two concepts may be fundamentally united around the resilience of function rather than state in microbial communities and the regularity in the relationship between environmental variation and a community's functional response. Furthermore, we posit that functional resilience is an intrinsic property of microbial communities and suggest that state changes in response to environmental variation may be a key mechanism driving functional resilience in microbial communities.

## Introduction

Microorganisms collectively exceed the biomass of all macrobiota on the planet (Whitman et al., 1998). Communities of microbes control the biogeochemical cycles upon which all macrobiota depend (Falkowski et al., 2008; Strom, 2008; Nazaries et al., 2013), and the role of microbial communities in shaping human health and physiology is also increasingly appreciated (Song et al., 2014a; Braundmeier et al., 2015; Lone et al., 2015; Sassone-Corsi and Raffatellu, 2015). Although natural microbial communities continually respond to perturbations (Konopka et al., 2014), their functioning can exhibit remarkable stability over time (Fuhrman et al., 2015), even under extreme environmental variation (Shade et al., 2012b; Lindemann et al., 2013). Comprehending the processes governing the responses of microbial communities to perturbation is critical both to ecologists concerned with predicting effects on ecosystem function (Hooper et al., 2005) and engineers designing communities for stable biotechnological processes (Lucas et al., 2015).

Though there is widespread interest in factors driving microbial community stability, the conceptual bases of stability measures, like resilience, are poorly defined. A report by the Community and Regional Resilience Institute (CARRI Report, 2013) summarized 47 definitions of resilience used in diverse scientific areas including engineering, ecology, sociology, economics, and psychology. Ambiguity is found even within disciplines: in ecology, resilience has been discussed alongside, and sometimes interchangeably with, ~70 other terms describing various stability measures (e.g., resistance, sustainability, and vulnerability; Grimm and Wissel, 1997). The conceptual variability in ecology surrounding stability, and resilience in particular, likely stems from system-specificity. Microbial communities span orders of magnitude in the diversity of their interacting components, from experimental or engineered systems to diverse natural communities. The metrics employed to evaluate each system's stability are largely idiosyncratic. The diversity of environments in which communities are investigated, the large array of functions of interest, and the range of research objectives concerning stability beg the question of whether a single definition of resilience can be universally applied across systems and scales for microbial communities.

Seeking an integrated concept applicable to all microbial communities, we herein compare engineering and ecological resilience and reconcile them by arguing that resilience is an intrinsic property of complex adaptive systems which, after perturbation, recover their system-level functions and interactions with the environment, rather than their endogenous state.

## Engineering and ecological concepts of resilience

**Figure 1:**
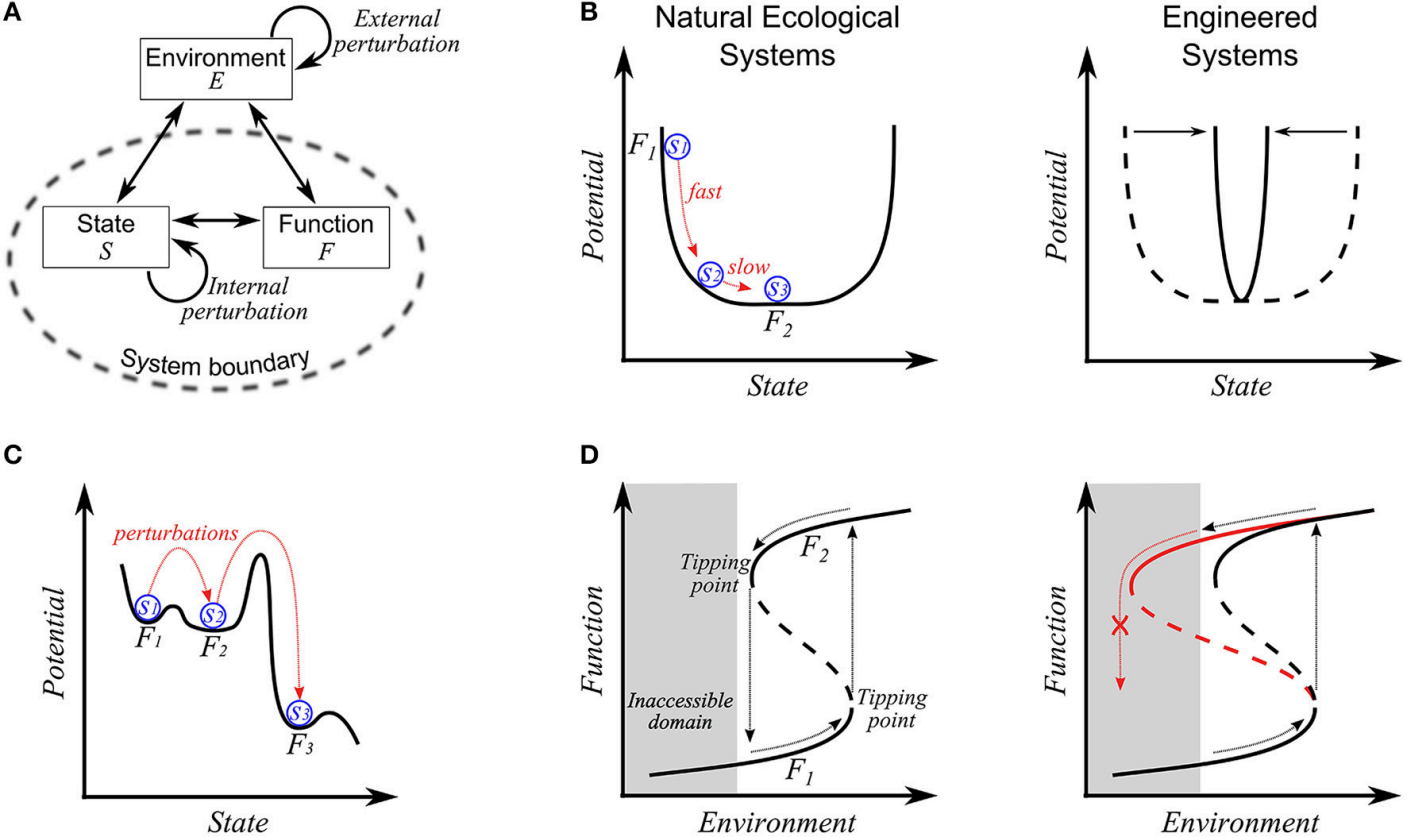
**(A)** A basic concept of stability-related properties. Against external and internal perturbations, the system adapts its state, which in turn may affect its functioning. Stability-related concepts such as resilience, resistance, and robustness are higher-order properties characterized by the system's response to imposed perturbations in terms of state, *S* or function *F*. In contrast, homeostasis is specifically confined to the system's ability to maintain or recover its state. **(B)** Resilience in compositionally complex, natural communities (left panel) and structurally simple, engineered consortia (right panel). On the left panel, the sequential changes from *S*_1_ to *S*_3_ and from *F*_1_ to *F*_2_, respectively, represent a temporal transition in state and function, right after disturbance. The linkage between function and state becomes weak on the flat bottom. The right panel shows the change of the profile from natural (dotted line) to engineered settings (solid line). **(C)** Stability landscape displaying the transition in state and function by perturbations. Three distinct wells denote domains of attractions (or regimes). The shift to a new regime may cause a significant change in state, but not in function (e.g., the transition between *F*_1_ and *F*_2_) or both in state and function (e.g., the transition between *F*_2_ and *F*_3_). **(D)** Hysteresis behaviors in microbial communities. The solid and dotted lines denote stable and unstable steady states, and the shaded area represents the infeasible domain that is inaccessible. Two stable branches (i.e., lower and upper) represent the reproducibly-observed relationship between environmental variables and community function. In the left panel, the community is initially on a lower, stable branch (i.e., *F*_1_). With the gradual change in an environmental variable, the community accordingly changes its composition and functional values; when it crosses a tipping point, the community undergoes abrupt changes in composition and function and arrives at an upper branch (i.e., *F*_1_). The original state and function are recovered when environmental variables decrease back through another drastic change in composition and function along the opposite direction on crossing another tipping point. In contrast, the right panel shows the case where recovery is impossible, e.g., due to the loss of member species or function during transition that results in a change in the shape of the hysteresis curve (as indicated by red line). In the case of repeated perturbation, member species or functions may be sequentially lost, and we expect the shape of the hysteresis curve to change incrementally over time in coordination with a community's compositional or functional drift.

Discussion of resilience in the literature often involves the related concepts of resistance and robustness. These stability-related properties are all concerned with the relationship between an imposed perturbation and a system's response (Figure 1A). Resilience has been broadly articulated as a system's *ability to recover* from disturbance. Diverse interpretations emerge, however, depending on what is considered “recovery” and how that recovery is quantified. In contrast, resistance has been defined with relatively less confusion, e.g., as the degree to which a system's state or function is insensitive to disturbance (Konopka et al., 2014). As a simple distinction, resilience is concerned with the system's ability to *recover* its function post-disturbance, while resistance is concerned with the system's ability to *maintain* its function against a perturbation. In these contexts, resilience (or resistance) denotes the degree to which the *quantitative* value of any function of interest is recovered to (or maintained at) an initial or reference condition. As illustrated elsewhere (Carpenter et al., 2001), systems may display significant resilience but not appreciable resistance and vice versa. In some cases, resilience has also been used as a synonym of robustness, described as the system's ability to maintain function post-disturbance (Levin and Lubchenco, 2008). Herein we consider robustness as a more general concept of stability that is comprised of resilience, resistance, and other complementary properties (Shade et al., 2012b), i.e., resilience and resistance are key components of a system's overall robustness.

The literature provides rich discussions of resilience, which can be subdivided into two categories: engineering and ecological concepts. As discriminated by Holling in his seminal paper (Holling, 1973), engineering resilience denotes the system's ability to recover its pre-perturbed equilibrium state as measured by the *rate* of return. The ecological concept considers a system's tolerance against disturbance without shifting to a new regime governed by fundamentally different processes and mechanisms (i.e., a domain of attraction), as quantified by the overall area of the domain of attraction or the depth of the basin. A domain of attraction represents a set of states converging to a given equilibrium point (such as S3 in Figure 1B, left panel). Both concepts of resilience have been invoked in microbial ecology. Faithful translation of the etymon of resilience, the Latin word *resalire* (literally, “to jump back”) (CARRI Report, 2013), interprets resilience as a property of elastic systems (as in physics/material sciences) that recover their original shape after disturbance. In this regard, Grimm and Calabrese (2011) equated elasticity with engineering resilience. Indeed, microbial communities share some features with elastic systems in that they sometimes undergo internal or structural deformation (e.g., changes of composition or gene expression patterns) under disturbance, yet eventually recover their original performance (e.g., recovery of microbe-driven biogeochemical processes after a forest fire; Tas et al., 2014). However, microbial communities lose elasticity (i.e., fail to recover) if the applied environmental stress exceeds a threshold, a phenomenon termed plastic deformation. The threshold across which plastic deformation occurs is variously called a tipping, yield, or bifurcation point (Veraart et al., 2012). Plastic deformation is better captured by the ecological concept of resilience. Obviously, microbial communities are neither perfectly elastic nor plastic, but are elasto-plastic systems. Thus, engineering and ecological concepts that reflect these aspects of microbial communities are complementary.

Variation in complexity between engineered and natural communities provides rationale for both the engineering and ecological concepts. Microbial communities in non-extreme natural settings typically have high compositional diversity and functional redundancy among species. As illustrated in Figure 1B (left panel), the community composition may vary over a certain range without affecting the function. The flat bottom of the profile implies *slow* dynamics in compositional change around a low-energy state, even in a rarely-perturbed or constant environment. Konopka et al. (2014) hypothesized that endogenous dynamics contribute to a community-level functional resilience. Relating resilience to the system's ability to persist within a domain of attraction after disturbance (i.e., ecological resilience), rather than the rate of return to the initial state, makes more sense in this circumstance. In contrast, microbial consortia with relatively lower diversity and functional redundancy should display a sharper curve (right panel of Figure 1B), meaning that (1) consortium-level function is maintained only at a specific compositional state and, (2) that the recovery to the original state after disturbance is relatively fast. In this second case, the rate of recovery may be a better measure of resilience (i.e., the engineering concept).

The distinction between engineering and ecological concepts of resilience fundamentally lies in their different foci: equilibrium vs. domain of attraction; numerical values of state variables vs. relationship between structure and function; rate of recovery after perturbation vs. ability to absorb the effect of disturbance (Grimm and Calabrese, 2011). In addition, we propose that the engineering and ecological concepts represent optimization for different objectives. Microbial consortia used for bioprocessing are, in essence, a set of biocatalysts that convert substrates into products. In well-designed bioreactors, where environmental conditions are tightly controlled, collapse across a tipping point may not be the major concern. Instead, rapid recovery after minor disturbances (i.e., engineering resilience) is critical to ensure consistent product quality and profit maximization. In contrast, with respect to natural communities with complex structure and dynamics, it would be of greater importance to proactively identify threats to ecosystem functioning by predicting how much additional stress a system can absorb without failure, a main concern in ecological resilience. In this regard, we add “maximizing system performance” vs. “preserving desirable system function” or “optimal control” vs. “monitoring and predicting” to the list of differences between engineering and ecological concepts.

The distinction in categorization of communities as “engineered” or “natural” becomes blurred in some cases. Wastewater treatment facilities (Adrados et al., 2014; Bernstein et al., 2014) and algal ponds (Park et al., 2011), for example, are engineered systems designed for a specific goal (i.e., water purification, biofuel production, or both) but are subject to environmental variations and influx of invasive species. Therefore, communities in natural and engineered environments, albeit structurally different, could be regarded as similar systems (subject to regular or episodic perturbations) to which an integrated concept of resilience could be applied. Toward this end, the theoretical underpinnings of the engineering and ecological concepts need to be understood more fundamentally.

## Resilience of composition, function, and the community-environment relationship

In principle, though the resilience of any system variable could be evaluated, a more fundamental question is at what level resilience is an intrinsic property of microbial communities (i.e., endogenous state or system function) and how state changes feed back to function. This issue has been extensively addressed by Kitano in regards to the robustness of biological systems (Kitano, 2004, 2007). He made a clear distinction between homeostasis and robustness, highlighting functional robustness as a property ubiquitously observed in biological systems, which often change in internal structure and mode of operation to preserve specific system functions against perturbation. We posit that Kitano's assertion, formulated to describe the behavior of single organisms, can be extended to describe the behavior of microbial communities. Thus, the translation of Röling et al. (2007) can be extended from enzymes in the cell to microbial species in a community.

In contrast to single organisms, microbial communities rarely have obvious *physical* boundaries that circumscribe the system, although locality of interactions is a hallmark of such communities (*sensu* Konopka) (Konopka, 2009). We could, however, assume a *hypothetical* boundary encompassing all interacting species and treat the community as a supra-organism, though, in practice, community boundaries are operationally defined. The individual components that constitute such a supra-organism can then, *in toto*, be considered to be the system's state variables, which include relative abundances of individual species, energy and material exchange across species (i.e., interspecies metabolic interactions), organismal gene expression patterns, and so forth. Community-level functions are then aggregate properties, such as the total growth rate and net uptake or production rates of metabolites. In practical terms, when discussing the resilience of microbial communities, it is the observer's task to unambiguously define both the system boundary and the function of interest measured in the community within that boundary. Theoretically, any variable or component of the community may be defined as the function of interest, and resilience of that function is necessarily relative to a specific perturbation. This is the “What to What?” approach detailed by Carpenter et al. (2001), which alleviates confusion of what quantified resilience signifies by supplying details regarding the system, the observed function, the cause and scale of perturbation, and the dimension of recovery.

Following Kitano, we argue that resilience is an intrinsic property of microbial communities that recover system-level functions after perturbation, instead of recovering a given endogenous state. Two related sub-hypotheses (SH) can be distinguished. SH1: the probability of a given community function being resilient (that is, capable of regaining its pre-perturbation value) is *hierarchical* (i.e., *p*_*comm*_ > *p*_*spec*_ > *p*_*subcell*_, where *p*_*comm*_, *p*_*spec*_, and *p*_*subcell*_ denote those probabilities of community-level, species-level, and subcellular variables, respectively), and SH2: the probability gap for functional resilience at different levels of organization is unequal (i.e., *p*_*comm*_ − *p*_*spec*_ < *p*_*spec*_ − *p*_*subcell*_). Together, these hypotheses imply that: (1) the intracellular state of an organism may change without affecting organism-level functions; (2) the function of organisms may change without impacting community-level functions; and (3) changes in community-level function happen at a relatively lower frequency than in organism-level function, which occur at lower frequency than intracellular state changes. While cases where this does not hold exist (e.g., Faith et al., 2011), examples for SH1 are commonly observed, e.g., changes in gene expression patterns are more susceptible to environmental variation than microbial growth rates and metabolic flux distributions (Ishii et al., 2007; Tang et al., 2009; Song et al., 2014b, 2015). Likewise, the resilience and functional stability in communities often arise as a result of significant compositional changes (Konopka et al., 2014). SH2 examples include cases where resilience of a community's composition and its function are closely linked, i.e., the gap between *p*_*comm*_ and *p*_*spec*_ is small. In a number of cases reported in the literature, the compositional resilience in microbial communities is shown to be comparable to functional resilience (Allison and Martiny, 2008; Shade et al., 2012a). Indeed, overall community function would be affected by compositional change even in cases where the role of one species can be replaced by other functionally-redundant members, unless the loss is not *quantitatively* compensated. It should be noted, however, that composition-function relationships depend on many factors including the specific function of interest, how many taxa perform the chosen function, and in what regime experimental data were collected. Thus, although we uphold the position that microbial communities are more likely to recover function, rather than composition, we regard compositional change as a potential governing mechanism of the whole system's resilience. To illustrate how community-level function can be maintained by changing internal state, we constructed a tutorial network representing a microbial community and simulated its responses to changes in environmental variables (Supplementary Material).

With a focus on the community-level performance, we restate the engineering concept of resilience as the rate of the system's recovery of its pre-perturbation *function*. This function-centered concept is linked to the ecological concept because the latter tolerates state changes that do not impact the overall function of interest. While exceptions exist, the tolerable magnitude of disturbance (the ecological concept) and the system dynamics (the engineering concept) are correlated around tipping points, i.e., systems nearing tipping points display peculiar behaviors such as slow recovery and magnified variations of state and functions (Dai et al., 2013). Thus, slow recovery of the ecosystem from disturbance may be an indicator of approaching a tipping point (Van Nes and Scheffer, 2007; Dai et al., 2012; Griffiths and Philippot, 2013; Dakos and Bascompte, 2014). The function-based view also expands the concept of a domain of attraction in ecological resilience. The stability landscape in Figure 1C, for example, shows three distinct domains of attraction. Suppose that the community performance at each regime is measured as *F*_1_, *F*_2_, and *F*_3_ (*F*_1_ ≈ *F*_2_ > *F*_3_), and its initial state is *S*_1_. In this case, we can consider the community to display resilience if the system returns (after perturbation) either to Regime 1 or to Regime 2. In this regard, Regimes 1 and 2 may be taken together as essentially the same functional domain.

Assessing resilience is challenging when microbial communities are subject to slow, chronic perturbation (e.g., climate change), or periodic environmental disturbances (e.g., temperature change during diel or seasonal cycles). Under such circumstances where the system's state, functions, and stability landscape are continually changing, it becomes unclear how to define a “pre-perturbed” condition for engineering resilience and a domain of attraction for ecological resilience. With respect to environmental variations that are much slower than the system's intrinsic dynamics, microbial communities may have sufficient time to adapt their endogenous state to the environment. We argue that this is the reason why some communities develop a stable relationship with their environment. Furthermore, the regularity in the relationship between environmental variation and the community's functional response is a fundamental aspect of resilience in that it applies to both fluctuating and constant environments. That is, microbial communities can be said to exhibit resilience as long as their functional interactions with the environment are reproducibly observed (Fuhrman et al., 2015). This idea of *relational resilience* (i.e., resilience of the relationship between environmental conditions and community function) provides fresh insight into system behavior at and around tipping points. On the left panel of Figure 1D, two stable branches of the hysteresis curve denote two unique relationships between the community function and environment. A tipping point is then defined as the condition across which a shift in the community-environment interaction occurs. Such non-linear hysteresis behavior has been observed in laboratory (Kim et al., 2012; Song and Ramkrishna, 2013) and ecological systems, e.g., coral reef-dominated vs. macroalgae-dominated state (Hughes et al., 2010); tropical forest vs. savanna vs. treeless state (Hirota et al., 2011). Early detection of nearby tipping points in ecosystems is therefore of practical importance before transition to an undesirable state and potentially permanent loss of critical functions. It may happen that key member species are lost during steady or abrupt change in environmental conditions. The resulting reduced diversity may lead to the change of stability landscape and subsequently the shape of the hysteresis curve so that catastrophic transition is very slow to recover or even *irreversible* (the right panel of Figure 1D). In this case, directly restoring lost members could be required to recover the systems' original functionalities. For example, pseudomembranous colitis is a condition in which *Clostridium difficile* dominates the gut microbiome after antibiotic treatment suppresses the normal, commensal microbiota. The measurable diversity of the *C. difficile*-dominated gut community is reduced compared with healthy controls (Song et al., 2013; Schubert et al., 2014), and large populations of *C. difficile* are difficult for normal commensal organisms to displace as these organisms enter the gut. Restoring members lost from the community through fecal transplantation has been effective in rapidly restoring the dysbiotic gut community to a more normal microbiome (Weingarden et al., 2015). Although restoring lost diversity imparts resilience to the community, similar approaches may be difficult for large-scale ecosystems, highlighting the importance of detecting diversity loss through continual monitoring and predicting the effects of environmental changes on community- and ecosystem-level responses.

## Conclusions and future research needs

Moving toward an integrated framework for understanding microbial community resilience, we propose reconciling concepts of engineering and ecological resilience through (1) consideration of microbial communities as systems that undergo both elastic and plastic deformation, and (2) defining resilience as the rate of recovery of a function of interest. Refocusing on the system's fundamental characteristics (such as the community-level functions and community-environment relationships) not only minimizes conceptual variation across different resilience definitions, but also provides a deeper understanding of the intrinsic community properties. In parallel, from a practical point of view, it is also of great importance to develop rational methods for quantifying microbial community resilience and predicting approaching tipping points.

Future research will need to address several important, unresolved issues—primarily, the identification of fundamental mechanisms responsible for microbial community resilience. For example, redundancy, diversity, and modularity are frequently advanced as mechanisms for robustness in complex systems (Kitano, 2004), but in some cases, and particularly in structurally simple consortia, they may not be directly related to resilience. The question remains: what unifying mechanisms impart resilience across both structurally simple and complex microbial communities? While the concept of networked buffering offers a potential mechanism (Whitacre and Bender, 2010; Konopka et al., 2014), rigorous analysis of microbial communities has yet to be performed. Another issue is the possible occurrence of trade-offs between system robustness (or resilience) and performance (Kitano, 2007) or trade-offs between robustness with respect to distinct perturbations (e.g., the conservation principle as discussed by Doyle and colleagues; Carlson and Doyle, 1999; Csete and Doyle, 2002). One major question is to what degree do resilience mechanisms identified for microbial communities overcome such trade-offs? Finally, the principles for structural organization of microbial communities as robust networks need to be further examined, as little is known about the general topological characteristics of microbial association networks and their relationships to resilience. Critical questions include: how does the compartmentalization of genes into a network of species affect the structural and higher-order properties of microbial communities; and are microbial community properties better understood as networks of species or networks of genes? Future research focusing on these issues will significantly advance our capability for the design, prediction, and control of microbial communities and maintenance of the critical ecosystem services they provide.

### Conflict of interest statement

The authors declare that the research was conducted in the absence of any commercial or financial relationships that could be construed as a potential conflict of interest.
