# Supplementary material for: Integrating Ecological and Engineering Concepts of Resilience in Microbial Communities
Source: Front Microbiol. 2015 Dec 1;6:1298. doi: 10.3389/fmicb.2015.01298 (PMC4664643; doi:10.3389/fmicb.2015.01298)
Supplement: Supplementary file 1 [file Presentation1.ZIP › Song_et_al_Frontiers2015_SuppInfo.pdf]

Integrating ecological and engineering concepts of resilience in microbial communities

Supplementary Information - Frontiers in Microbiology - 2015

#####

- Hyun-Seob Song[1], Ryan S. Renslow[2], Jim K. Fredrickson[1], Stephen R. Lindemann[1**]
- [1]Biological Sciences Division, Fundamental and Computational Sciences Directorate, Pacific Northwest National Laboratory, Richland, WA, USA
- [2]Environmental Molecular Sciences Laboratory, Pacific Northwest National Laboratory, Richland, WA, USA
- [**] Correspondence: Stephen R. Lindemann, Biological Sciences Division, Fundamental and Computational Sciences Directorate, Pacific Northwest National Laboratory, 902 Battelle Boulevard, MSIN: J4-18, Richland, WA 99352, USA e-mail: stephen.lindemann@pnnl.gov

Exploring the relationship between Environment, State, and Function through a simple network

To demonstrate the relationship between environment, state and funciton, a simple network was constructed with the goal of supplementaing key ideas from the manuscript. The network consists of 4 nodes with 2 environmental variables, 6 state variables, and 1 functional variable.

All possibles environment-state-function solutions are controlled by 5 variables: the 2 environemntal variables and 3 network variables.

Import Python modules and define classes required for this iPython Notebook to run:

In [1]:
```
%matplotlib inline
from IPython.display import clear_output
import csv
import random
from random import sample
import numpy as np
import matplotlib.pyplot as plt
from mpl_toolkits.mplot3d.axes3d import Axes3D
from matplotlib  import cm
import networkx as nx
class ListTable(list):
    def _repr_html_(self):
        html = ["<table>"]
        for row in self:
            html.append("<tr>")
            for col in row:
                html.append("<td>{0}</td>".format(col))
            html.append("</tr>")
        html.append("</table>")
        return ''.join(html)
```

The Networkx module is used to define the network. The Networkx module was developed by Aric Hagberg and Pieter Swart of the DOE Los Alamos National Laboratory and Daniel Schult of Colgate University.

Citation: *Aric A. Hagberg, Daniel A. Schult and Pieter J. Swart, "Exploring network structure, dynamics, and function using NetworkX", in Proceedings of the 7th Python in Science Conference (SciPy2008), Gäel Varoquaux, Travis Vaught, and Jarrod Millman (Eds), (Pasadena, CA USA), pp. 11--15, Aug 2008*

In [2]:

```
N = nx.Graph()
N.add_node('Env1',pos=(-2.2,0))
N.add_node('Env2',pos=(1,-2.2))
N.add_node('A',   pos=(-1,0))
N.add_node('B',   pos=(0,1))
N.add_node('C',   pos=(0,-1))
N.add_node('D',   pos=(1,0))
N.add_node('E',   pos=(2.2,1))
N.add_node('Func',pos=(2.2,0))


N.add_edge('Env1', 'A',weight=4)
N.add_edge('A', 'B',weight=4)
N.add_edge('A', 'C',weight=4)
N.add_edge('B', 'C',weight=4)
N.add_edge('B', 'E',weight=4)
N.add_edge('C', 'D',weight=4)
N.add_edge('B', 'D',weight=4)
N.add_edge('Env2', 'D',weight=4)
N.add_edge('D', 'Func',weight=4)

pos=nx.get_node_attributes(N,'pos')
weights = [N[u][v]['weight'] for u,v in N.edges()]

fig = plt.figure(figsize=(10,8))

def drawnetwork(weights):
    circle = plt.Circle((0, 0), radius=1.5,ls='dashed', fc='#905FA9',alpha =
1,linewidth=3,zorder=0)
    plt.gca().add_patch(circle)
    nx.draw_networkx_nodes(N, pos, nodelist=['Env1','Env2'],node_color='#3D985
8',node_size=3000,linewidths=3)
    nx.draw_networkx_nodes(N, pos, nodelist=['A','B','C','D','E'],node_colo
r='#B04A3C',node_size=3000,linewidths=3)
    nx.draw_networkx_nodes(N, pos, nodelist=['Func'],node_color='w',node_size=3
000,linewidths=3)
    nx.draw_networkx_edges(N, pos, edge_color='k', width=weights)
    nx.draw_networkx_labels(N,pos,font_size=20,zorder=10)
    plt.axis('off')
    plt.axis('equal')
    #plt.savefig("network.png")

drawnetwork(weights)
plt.text(-1.7,.1,s=r'$E_1$',horizontalalignment='center',fontsize=20)
plt.text(0.8,-1.7,s=r'$E_2$',horizontalalignment='center',fontsize=20)
plt.text(-.6,.6,s=r'$S_1$',horizontalalignment='center',fontsize=20)
plt.text(-.5,-.3,s=r'$S_2$',horizontalalignment='center',fontsize=20)
plt.text(-.2,0,s=r'$S_3$',horizontalalignment='center',fontsize=20)
plt.text(.6,.6,s=r'$S_4$',horizontalalignment='center',fontsize=20)
plt.text(.5,-.3,s=r'$S_5$',horizontalalignment='center',fontsize=20)
plt.text(1.5,1.1,s=r'$S_6$',horizontalalignment='center',fontsize=20)
plt.text(1.7,.1,s=r'$F_1$',horizontalalignment='center',fontsize=20)
plt.show()
```

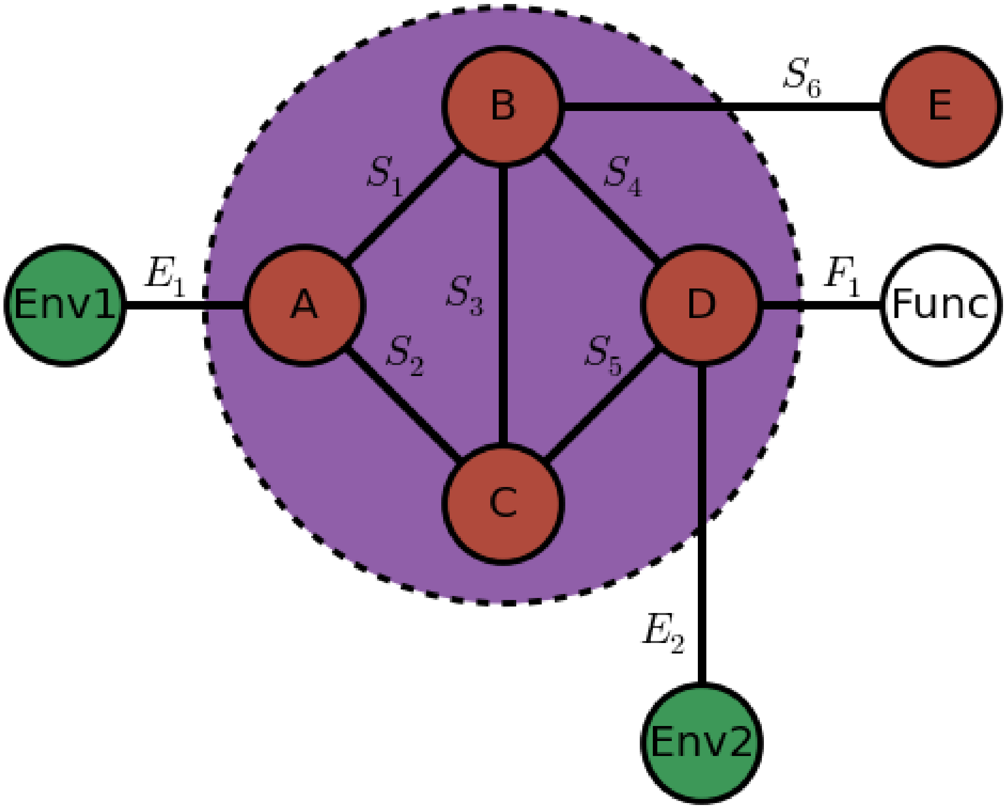

Supp Figure 1. Simple network with two environmental variables and one functional variable.

Define the stoichiometric relationship and flux direction between nodes:

In [3]:
```
def calcnetwork(inputs):
    [E1,E2,net1,net2,net3] =  inputs
    S1 = E1*net1
    S2 = E1*(1-net1)
    S3 = S1*net2*0.5
    S4 = (1-net2)*S1*net3*2
    S5 = S2+S3
    S6 = S1*(1-net2)*(1-net3)*0.5
    F1 = 2*E2+S4+S5
    return F1, S1, S2, S3, S4, S5, S6
```

Define a flux balance on nodes A through D to ensure a balanced network:

In [4]:
```
def balancenetwork(inputs):
    F1, S1, S2, S3, S4, S5, S6 = calcnetwork(inputs)
    nodeA = E1-S1-S2
    nodeB = S1-2*S3-0.5*S4-2*S6
    nodeC = S2+S3-S5
    nodeD = 2*E2+S4+S5-F1

    if abs(nodeA+nodeB+nodeC+nodeD) <= 10**(-10):
        status = 'Network Balanced!'
    else:
        status = 'Network is not balanced...'
    print(status)
    return nodeA+nodeB+nodeC+nodeD
```

Define a set of test network input parameters to calculate initial function values and check if network is balanced. Assume that E1 and E2 range from 0 to 100, and all network variables range from 0-100%.

In [5]:
```
E1 = 10
E2 = 10
net1 = 0.25
net2 = 0.75
net3 = 0.50
inputs = [E1,E2,net1,net2,net3]
print('F1 is',calcnetwork(inputs)[0])
balancetest = balancenetwork(inputs)
```

```
F1 is 29.0625
Network Balanced!
```

Now we can explore the full range of the environment, state, and function relationships. The data is saved as a csv for recall and use later. The *sample* function can be used to randomly sample values in the full range of environmental and network variables; or the *range* function, currently in comments, may be used to sample regularly spaced values of the environmental and network variables. For large sample sizes, the output will flash "Network Balanced!" while running, and then "Complete!" when it is finished.

In [6]:
```
f = open('networkdata.csv', 'w', newline='')
fileWriter = csv.writer(f)
fileWriter.writerow(['E1','E2','Net1','Net2','Net3','S1','S2','S3','S4','S5','S
6','F1','Balance'])

for E1 in sample(range(101), 7):#range(0,101,20):
    for E2 in sample(range(101), 7):#range(0,101,20):
        for net1L in sample(range(101), 7):#range(0,101,20):
            for net2L in sample(range(101), 7):#range(0,101,20):
                for net3L in sample(range(101), 7):#range(0,101,20):
                    net1 = net1L/100
                    net2 = net2L/100
                    net3 = net3L/100
                    inputs = [E1,E2,net1,net2,net3]
                    F1, S1, S2, S3, S4, S5, S6 = calcnetwork(inputs)
                    baltest = balancenetwork(inputs)

                    fileWriter.writerow([E1,E2,net1,net2,net3,S1,S2,S3,S4,S5,S
6,F1,baltest])
                    clear_output()
print('Complete!')
```

```
Complete!
```

To visualize the relationship between environment, state, and function, principal component analysis is used for dimension reduction. The final three dimensions are called Environment, State, and Function.

In [32]:
```
networkdata = np.genfromtxt('network_data_small.csv', delimiter=',', skip_heade
r=1) #'network_data_small.csv'
statedata = networkdata[:,2:11]
envdata = networkdata[:,0:2]
funcdata = networkdata[:,11]

state_cov_matrix = np.cov(statedata.T)
state_eig_value, state_eig_vector = np.linalg.eig(state_cov_matrix)
state_matrix_w = state_eig_vector[:,1]
statetransformed = state_matrix_w.T.dot(statedata.T)
statetransformed = (statetransformed-min(statetransformed))/(max(statetransform
ed)-min(statetransformed))

env_cov_matrix = np.cov(envdata.T)
env_eig_value, env_eig_vector = np.linalg.eig(env_cov_matrix)
env_matrix_w = env_eig_vector[:,1]
envtransformed = env_matrix_w.T.dot(envdata.T)
envtransformed = (envtransformed-min(envtransformed))/(max(envtransformed)-mi
n(envtransformed))

funcdata = (funcdata-min(funcdata))/(max(funcdata)-min(funcdata))
```

Generate a 3D plot of environment, state and function:

In [33]:

```
fig = plt.figure(figsize=(10,10))

ax = fig.add_subplot(111, projection='3d')
ax.scatter(statetransformed, envtransformed, funcdata,s=(funcdata**1.5)*300,c=s
tatetransformed, marker = 'o', cmap = cm.jet )
ax.set_title("Relationship between Environment, State, and Function",fontsize=2
0)
ax.set_xlabel("State",fontsize=16)
ax.set_ylabel("Environment",fontsize=16)
ax.set_zlabel("Function",fontsize=16)
ax.grid(True,linestyle='-',color='0.75')
ax.set_xlim3d(0,1)
ax.set_ylim3d(0,1)
ax.set_zlim3d(0,1)
plt.show()
```

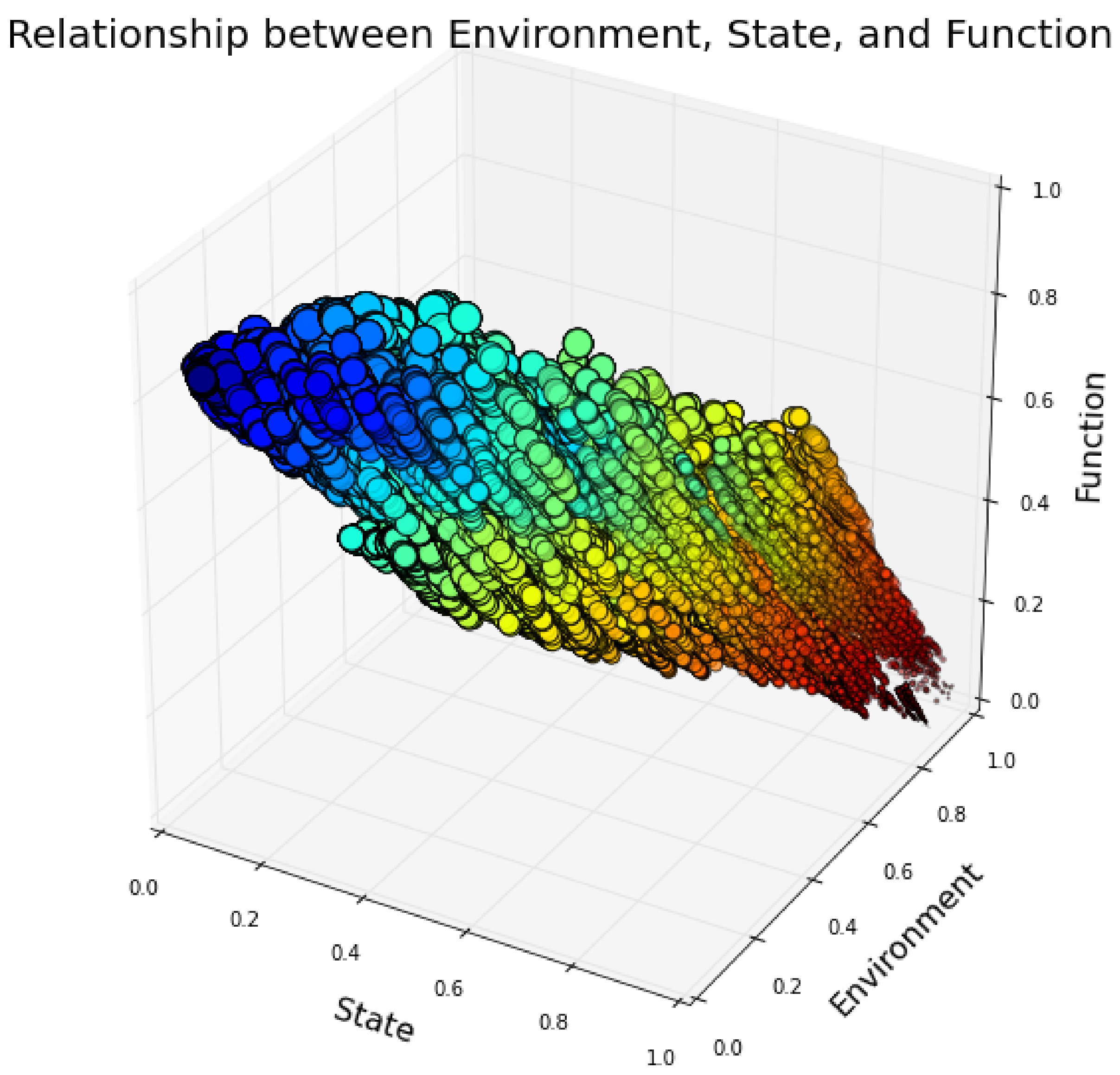

Supp Figure 2. Relationship between the environment, state, and function in the simple network.

Next section generates a 2D plot of environment and state

In [34]:
```
fig = plt.figure(figsize=(16,8))
ax1 = plt.subplot(1,2,1)
ax1.set_title("Relationship between Environment and State",fontsize=20)
ax1.set_xlabel("Environment",fontsize=16)
ax1.set_ylabel("State",fontsize=16)
ax1.grid(True,linestyle='-',color='0.75')
plt.axis([0,1,0,1])
ax1.scatter(envtransformed,statetransformed,s=(funcdata**1.5)*300,c=statetransf
ormed, marker = 'o', cmap = cm.jet );
```

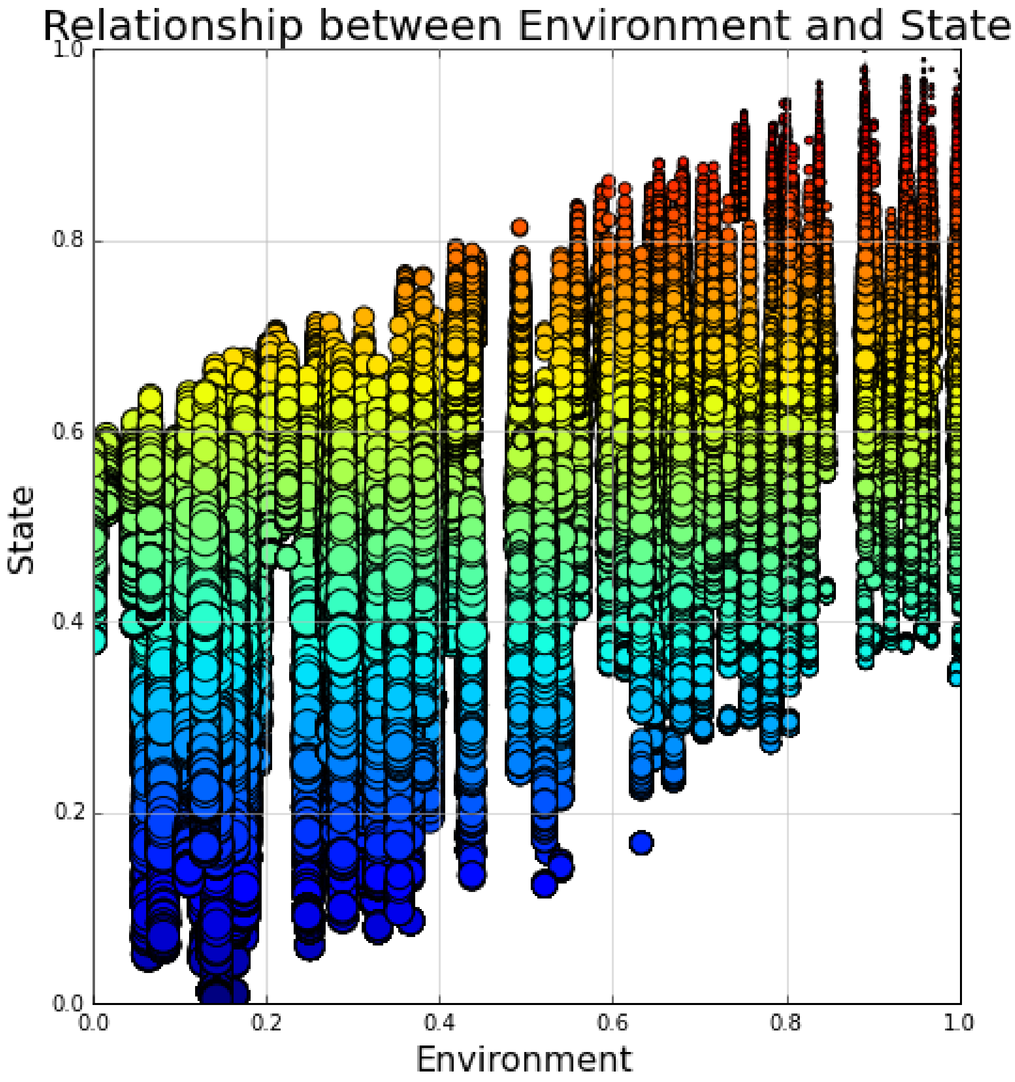

Supp Figure 3. Relationship between environment and state in the simple network.

Generate a 2D plot of State and Function, showing two different Function outputs. This plot highlights the fact that function may remain the same even while the state is changing. For example, State I, II, and III all produce Function A.

In [35]:

```
filter_FuncA = np.logical_and(funcdata>0.83,funcdata<0.84)
filter_FuncB = np.logical_and(funcdata>0.15,funcdata<0.16)

fig = plt.figure(figsize=(8,8))
ax = plt.subplot(1,1,1)
ax.set_title("Relationship between State and Function",fontsize=20)

ax.set_xlabel("State",fontsize=16)
ax.set_ylabel("Function",fontsize=16)
ax.grid(True,linestyle='-',color='0.75')
plt.axis([0,1,0,1])
ax.text(0.25, 0.88, 'Function A',fontsize=14,horizontalalignment='center')
ax.text(0.77, 0.19, 'Function B',fontsize=14,horizontalalignment='center')
ax.text(0.05, 0.74, 'State\nI',fontsize=14,horizontalalignment='center')
ax.text(0.25, 0.74, 'State\nII',fontsize=14,horizontalalignment='center')
ax.text(0.45, 0.74, 'State\nIII',fontsize=14,horizontalalignment='center')
ax.text(0.60, 0.07, 'State\nIV',fontsize=14,horizontalalignment='center')
ax.text(0.77, 0.07, 'State\nV',fontsize=14,horizontalalignment='center')
ax.text(0.94, 0.07, 'State\nIV',fontsize=14,horizontalalignment='center')
ax.scatter(np.concatenate([statetransformed[filter_FuncA],statetransformed[filt
er_FuncB]]),np.concatenate([funcdata[filter_FuncA],funcdata[filter_FuncB]]),c=n
p.concatenate([statetransformed[filter_FuncA],statetransformed[filter_Func
B]]),s=(np.concatenate([funcdata[filter_FuncA],funcdata[filter_FuncB]])**1.5)*3
00, marker = 'o', cmap = cm.jet)
plt.show()
```

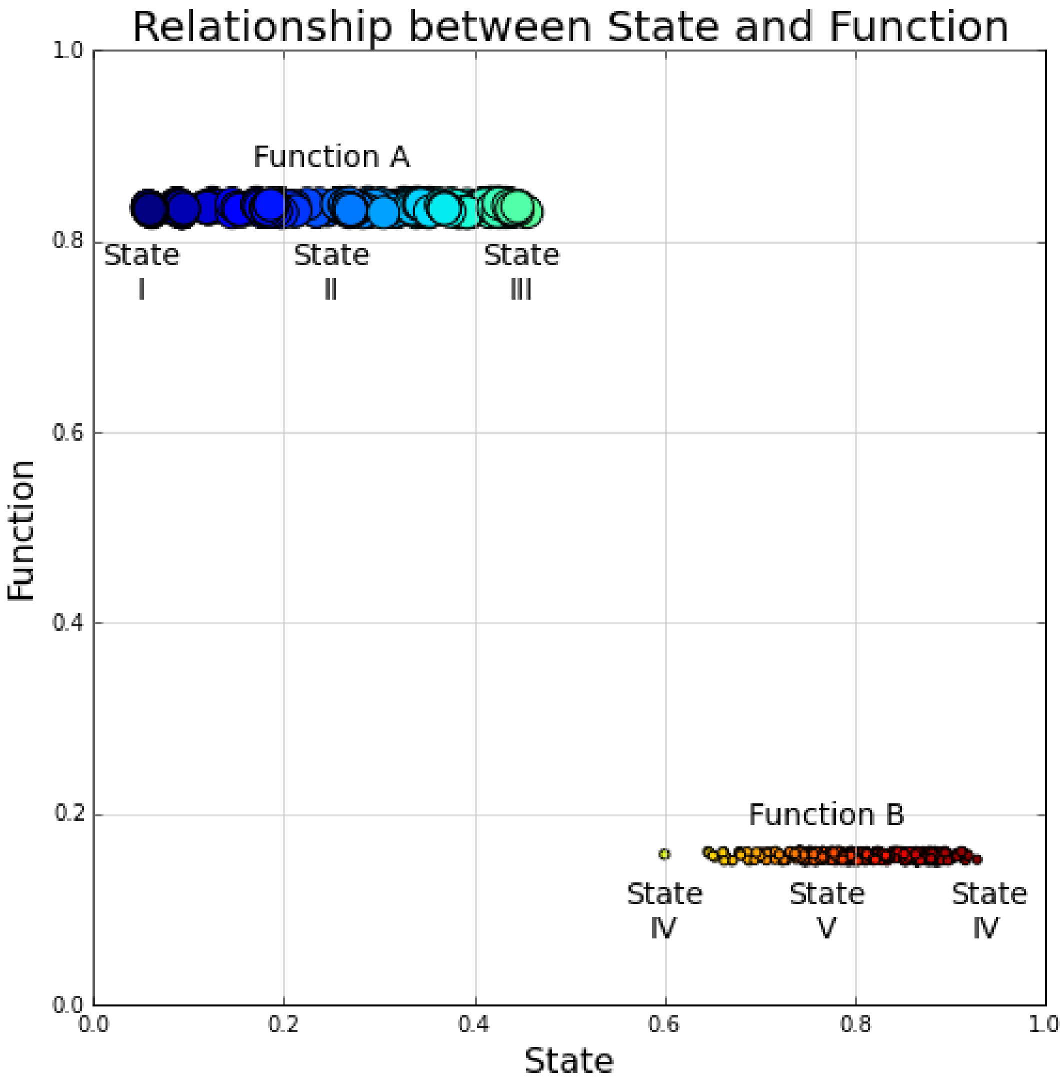

Supp Figure 4. Relationship between state and function in the simple network. Function may remain the same (A or B), even while the state may change greatly (states I through IV).

We can visualize the different states for while function remains the same. The thickness of each network connection is scaled to represent the magnitude of flux relative to the maximum flux possible through each connection.

In [36]:

```
fig = plt.figure(figsize=(25,7))
fig.suptitle("Function A",y=1.05, fontsize=30)
plt.subplot(131)
plt.title("State I",fontsize=20)
weights=[.81,1.0,.72,.09,.25,.67,.14,.21,.72]
drawnetwork([x * 40 for x in weights])

plt.subplot(132)
plt.title("State II",fontsize=20)
weights=[.81,1.0,.72,.09,.40,.65,.02,.29,.72]
drawnetwork([x * 40 for x in weights])

plt.subplot(133)
plt.title("State III",fontsize=20)
weights=[.81,1.0,.15,.66,.05,.55,.01,.68,.72]
drawnetwork([x * 40 for x in weights])
plt.show()

fig = plt.figure(figsize=(25,7))
fig.suptitle("Function B",y=1.05, fontsize=30)
plt.subplot(131)
plt.title("State IV",fontsize=20)
weights=[.29,.13,.28,.01,.09,.13,.06,.06,.15]
drawnetwork([x * 40 for x in weights])

plt.subplot(132)
plt.title("State V",fontsize=20)
weights=[.30,.15,.26,.04,.18,.11,.01,.13,.14]
drawnetwork([x * 40 for x in weights])

plt.subplot(133)
plt.title("State VI",fontsize=20)
weights=[.29,.13,.04,.25,.02,.08,.01,.26,.14]
drawnetwork([x * 40 for x in weights])
plt.show()
```

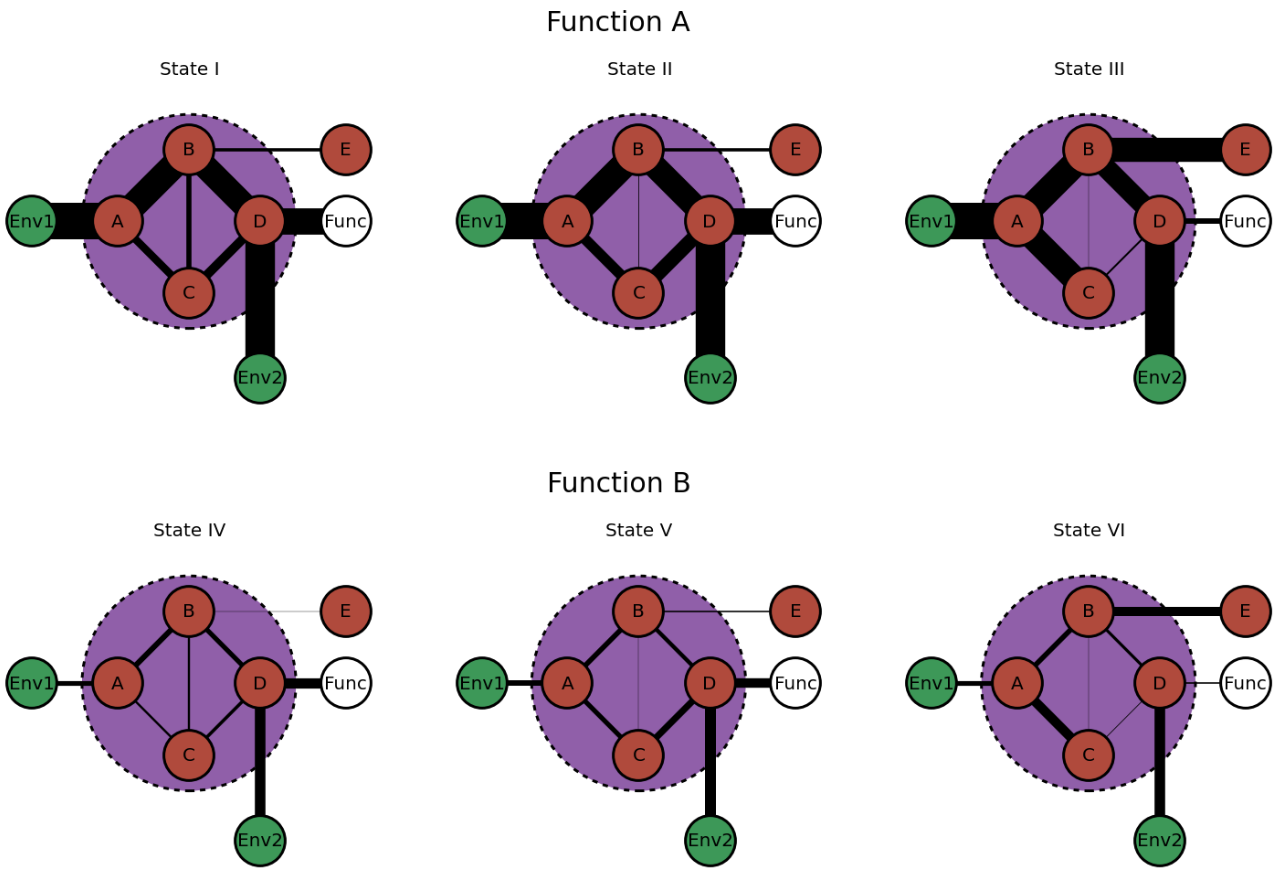

Supp Figure 5. Visualization of the simple network for six different states, which generate two different functions (A and B).

A sensitivity analysis on function is performed by measuring relative function change after a 20% change on each input variable. The sensitivity of function to each input variable depends on the state.

In [38]:

```
def sensitivitynetwork(funcstat,env1,env2,net1,net2,net3):
    inputs = [env1,env2,net1,net2,net3]
    inputs_env1s = [env1*0.8,env2,net1,net2,net3]
    inputs_env2s = [env1,env2*0.8,net1,net2,net3]
    inputs_net1s = [env1,env2,net1*0.8,net2,net3]
    inputs_net2s = [env1,env2,net1,net2*0.8,net3]
    inputs_net3s = [env1,env2,net1,net2,net3*0.8]
    env1_sens = round(abs((calcnetwork(inputs_env1s)[0]-calcnetwork(inputs)
[0])/(calcnetwork(inputs)[0])/.2),2)
    env2_sens = round(abs((calcnetwork(inputs_env2s)[0]-calcnetwork(inputs)
[0])/(calcnetwork(inputs)[0])/.2),2)
    net1_sens = round(abs((calcnetwork(inputs_net1s)[0]-calcnetwork(inputs)
[0])/(calcnetwork(inputs)[0])/.2),2)
    net2_sens = round(abs((calcnetwork(inputs_net2s)[0]-calcnetwork(inputs)
[0])/(calcnetwork(inputs)[0])/.2),2)
    net3_sens = round(abs((calcnetwork(inputs_net3s)[0]-calcnetwork(inputs)
[0])/(calcnetwork(inputs)[0])/.2),2)
    return [funcstat,env1_sens,env2_sens,net1_sens,net2_sens,net3_sens]

table = ListTable()
table.append(['','Env1','Env2','Net1','Net2','Net3'])

table.append(sensitivitynetwork('Func A: State I',81,100,0.89,0.42,0.04))
table.append(sensitivitynetwork('Func A: State II',81,100,0.89,0.42,0.04))
table.append(sensitivitynetwork('Func A: State III',81,100,0.89,0.54,0.91))
table.append(sensitivitynetwork('Func B: State IV',29,13,0.97,0.47,0.37))
table.append(sensitivitynetwork('Func B: State V',30,15,0.86,0.29,0.04))
table.append(sensitivitynetwork('Func B: State VI',29,13,0.15,0.51,0.02))
table
```

Out[38]:

	Env1	Env2	Net1	Net2	Net3
Func A: State I	0.12	0.88	0.24	0.06	0.01
Func A: State II	0.12	0.88	0.24	0.06	0.01
Func A: State III	0.31	0.69	0.03	0.18	0.21
Func B: State IV	0.42	0.58	0.24	0.07	0.25
Func B: State V	0.24	0.76	0.52	0.08	0.04
Func B: State VI	0.5	0.5	0.06	0.02	0.0

Supp Table 1. Sensitivity analysis results for all six states. The state affects the sensitivy of the function to changes in each variable.
